# Supplementary figures and images for: The 10-year course of mental health, quality of life, and exile life functioning in traumatized refugees from treatment start
Source: PLoS One. 2020 Dec 31;15(12):e0244730. doi: 10.1371/journal.pone.0244730 (PMC7775068; doi:10.1371/journal.pone.0244730)

### S1 Smooth Curves to check for non-linearity

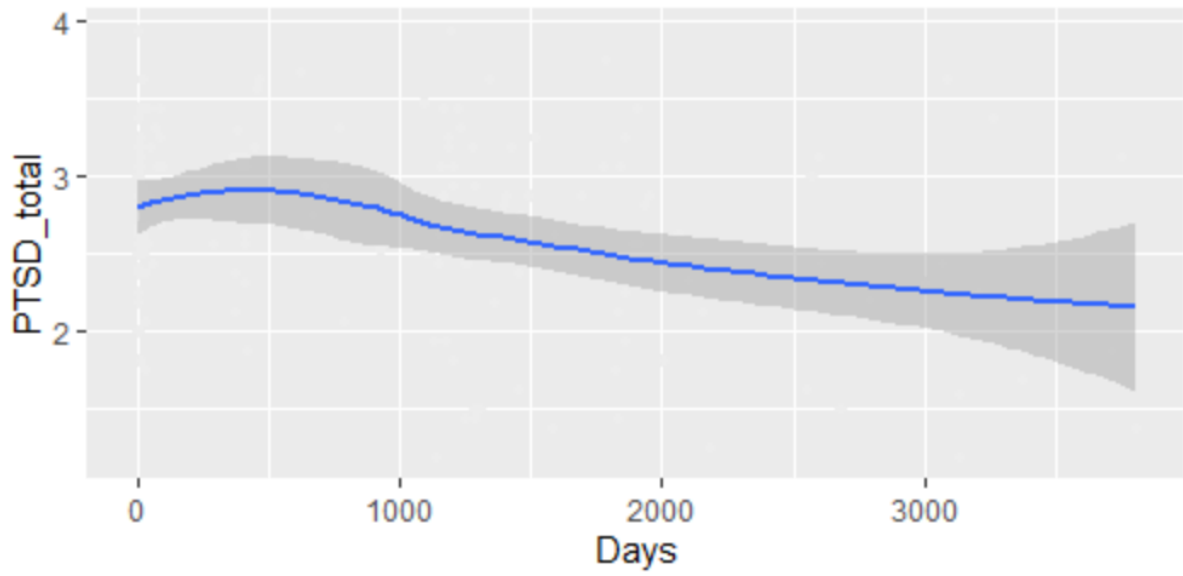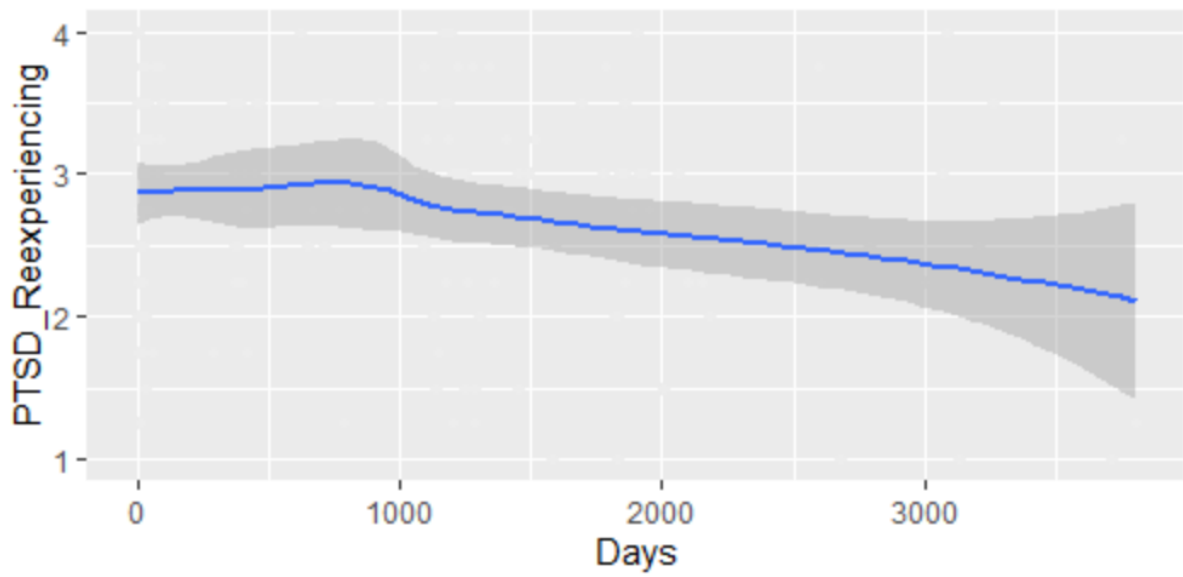

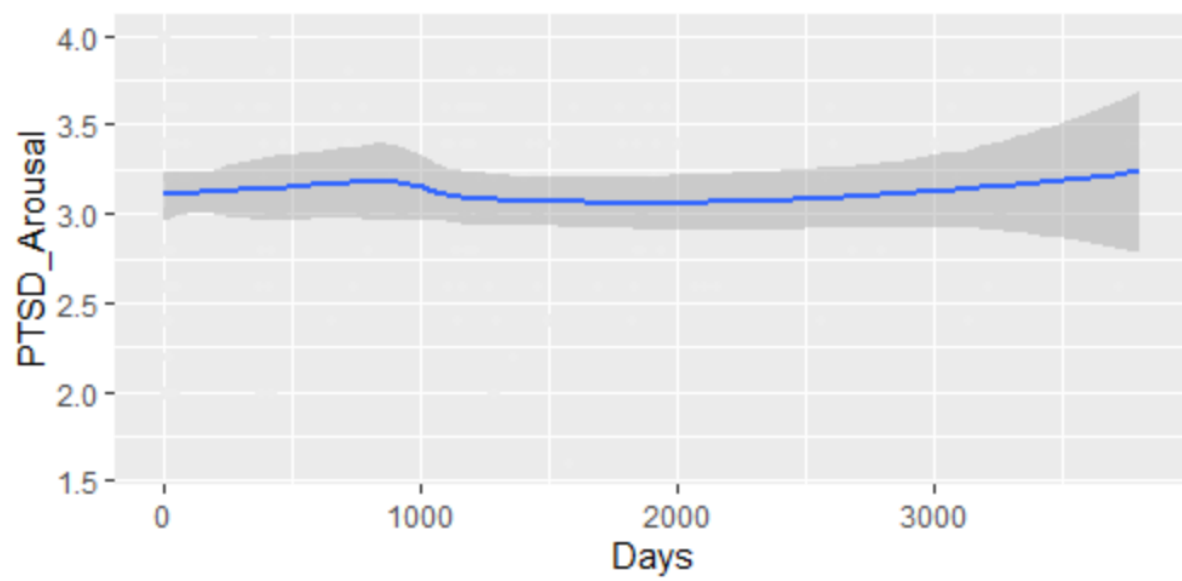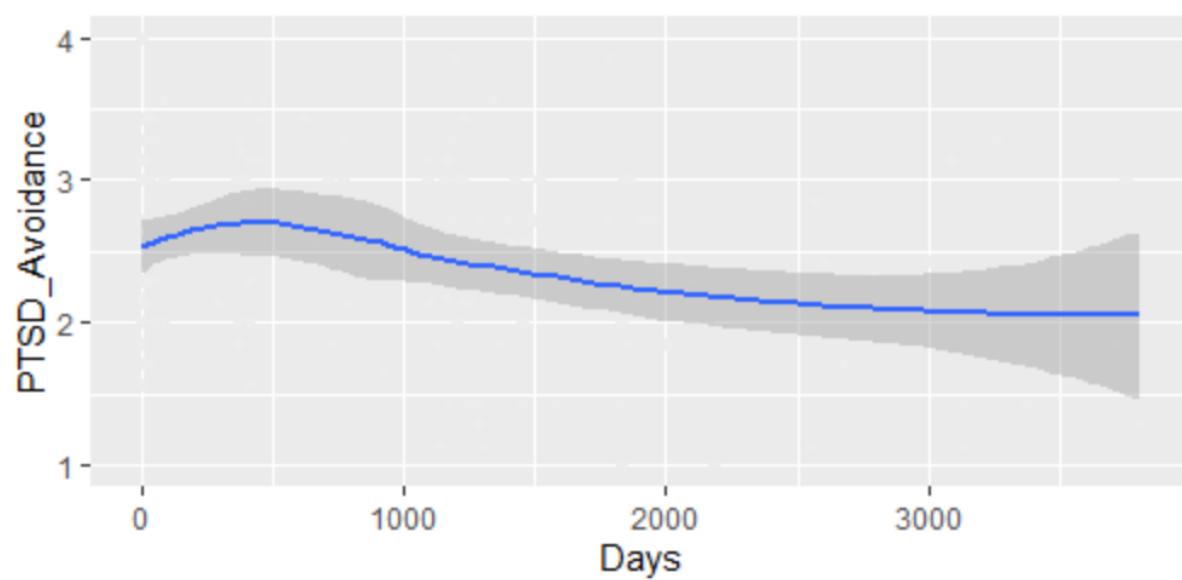

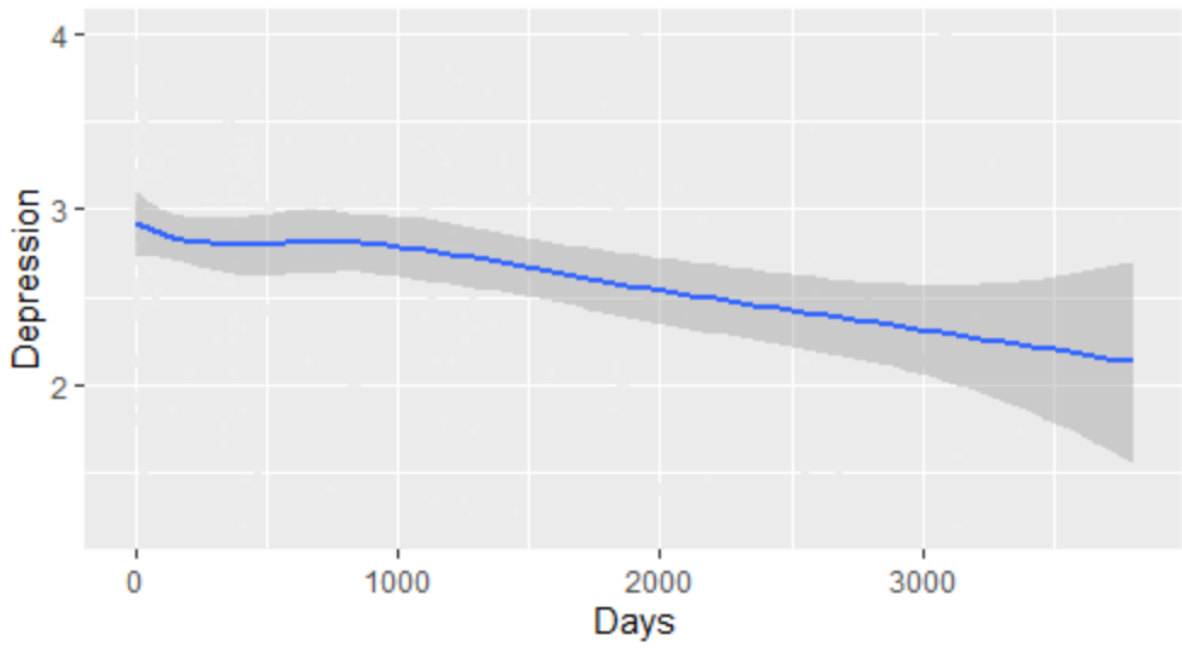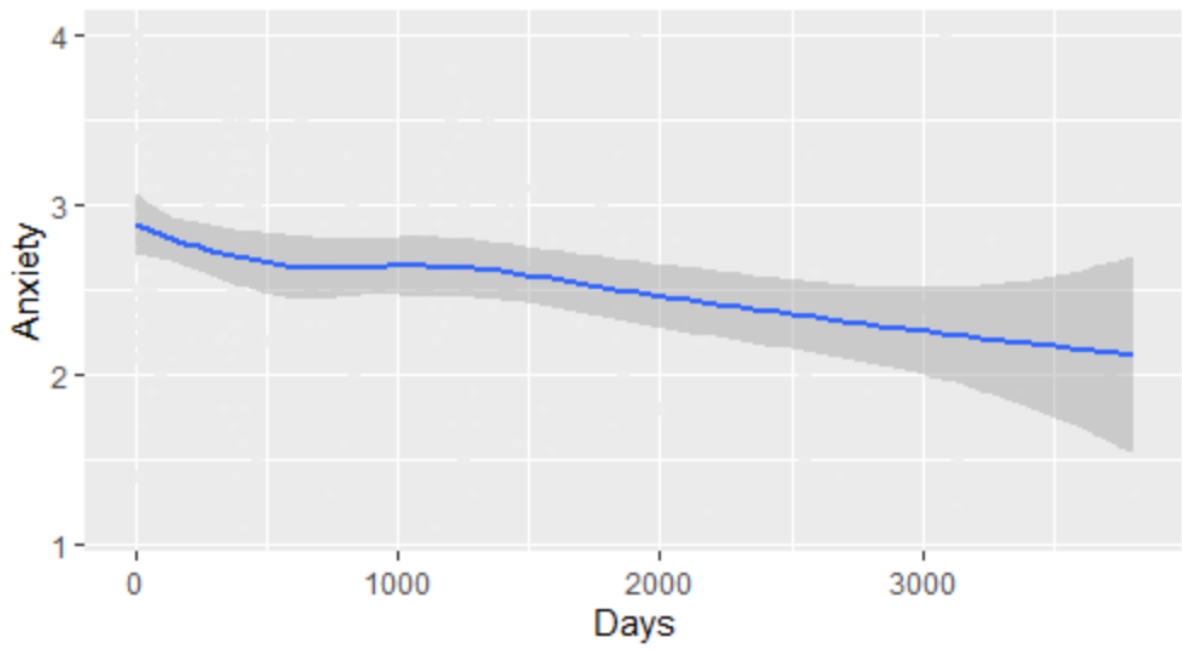

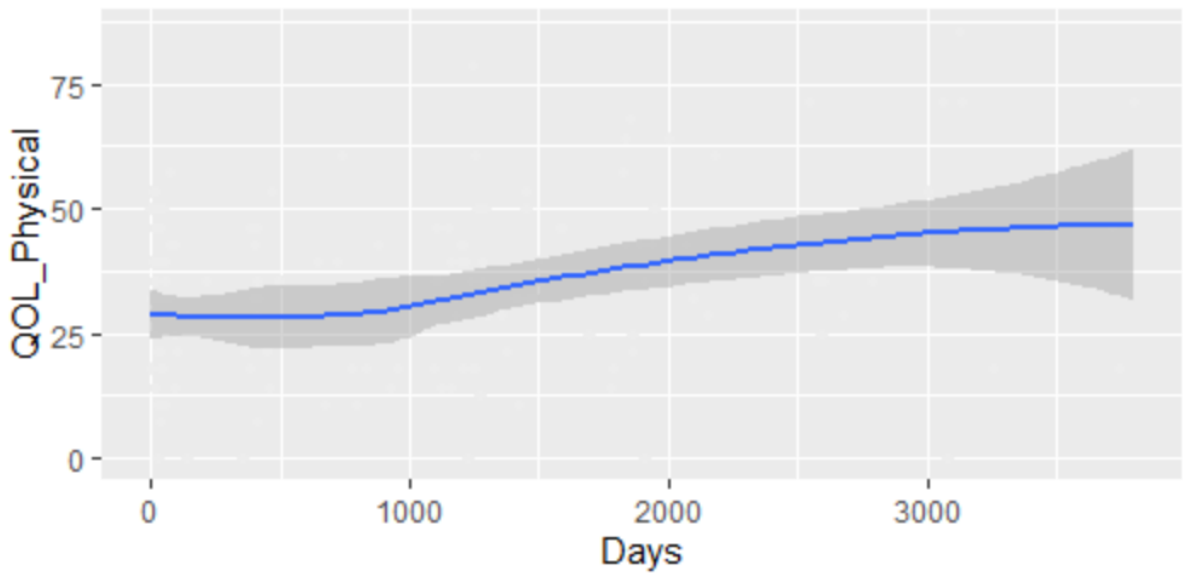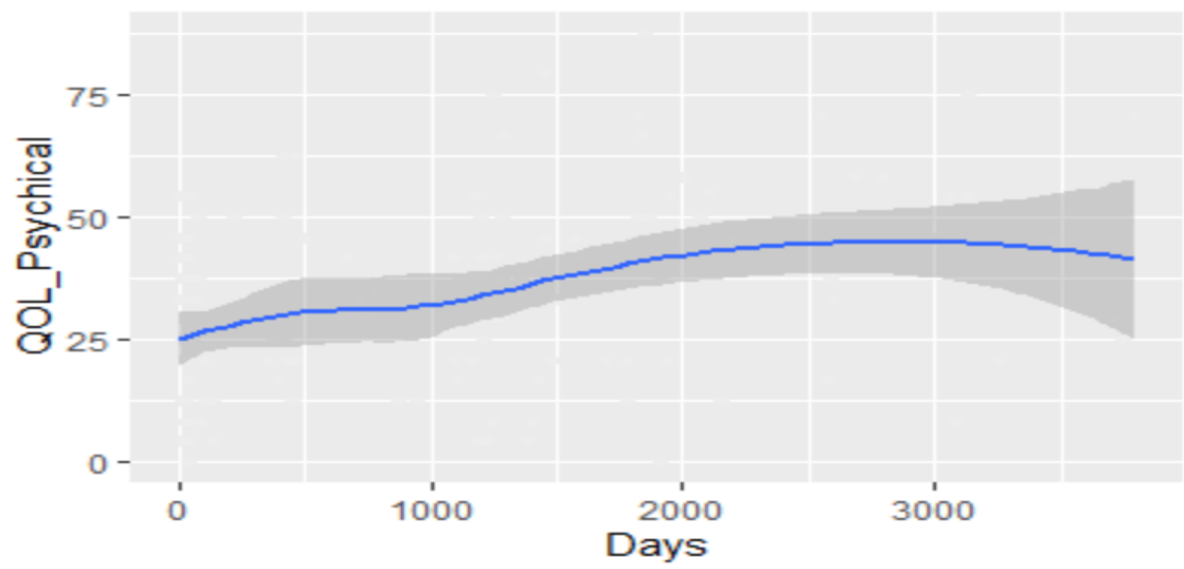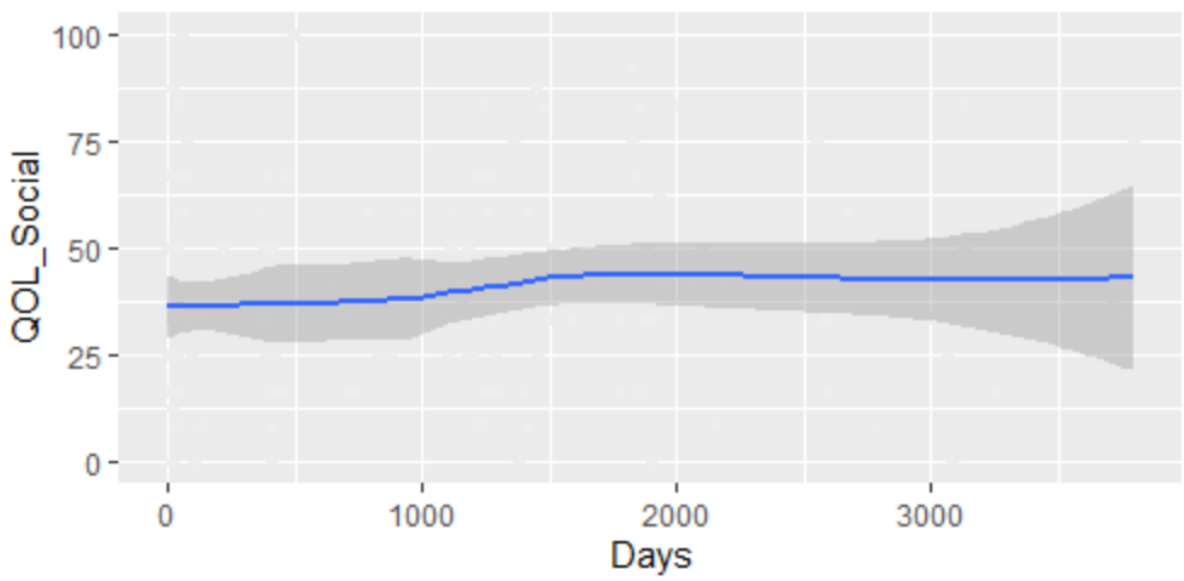

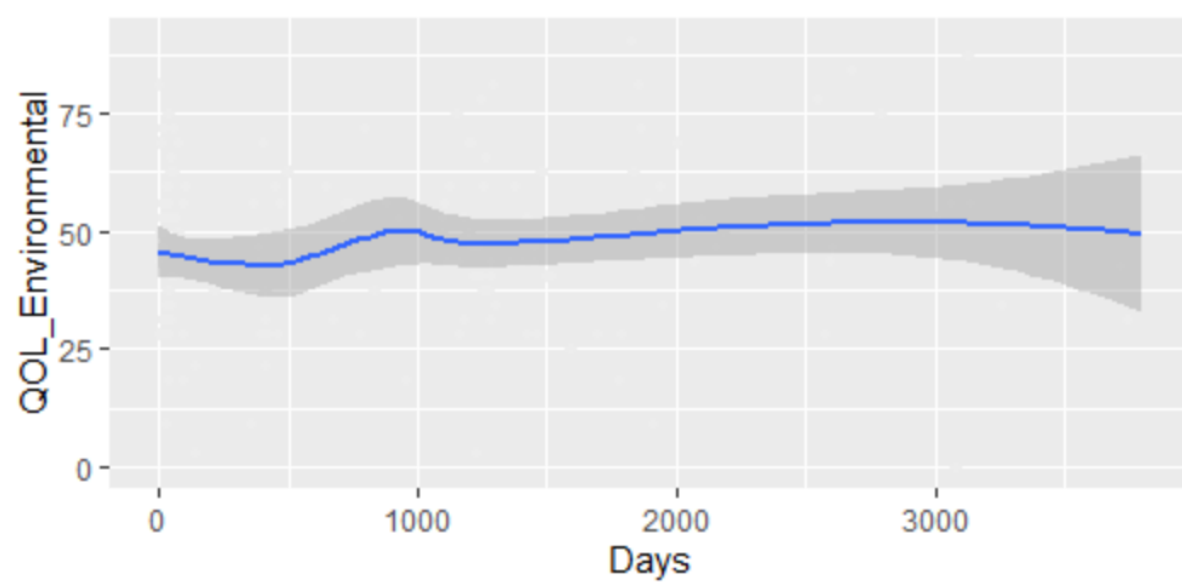

Supplement: S1 Fig — (PDF) [file pone.0244730.s001.pdf]
