# Supplementary material for: The 10-year course of mental health, quality of life, and exile life functioning in traumatized refugees from treatment start
Source: PLoS One. 2020 Dec 31;15(12):e0244730. doi: 10.1371/journal.pone.0244730 (PMC7775068; doi:10.1371/journal.pone.0244730)
Supplement: Attachment — Submitted filename: Response to reviewers.docx [file pone.0244730.s006.docx]

PONE-D-20-12948 PLOS ONE

The 10-Year Course of Mental Health, Quality of Life, and Exile Life Functioning in Traumatized Refugees from Treatment Start

# **Response to reviewers**

*(Our replies/comments in italics)*

## **1. Response to academic editor**

1. Please ensure that your manuscript meets PLOS ONE's style requirements, including those for file naming. The PLOS ONE style templates can be found at

<https://journals.plos.org/plosone/s/file?id=wjVg/PLOSOne_formatting_sample_main_body.pdf> and

<https://journals.plos.org/plosone/s/file?id=ba62/PLOSOne_formatting_sample_title_authors_affiliations.pdf>

*We have rechecked PLOS ONE’s style requirements and made the ensuing corrections. I hope the style is now correct.*

2. Please provide additional details regarding participant consent. In the ethics statement in the Methods and online submission information, please ensure that you have specified (a) whether consent was informed and (b) what type you obtained (for instance, written or verbal, and if verbal, how it was documented and witnessed). If the need for consent was waived by the ethics committee, please include this information.

*Under Design in the Methods section, we added the last sentences (here in italics) to the following passage: “The study was approved by the Norwegian South-East Regional Committee for Medical and Health Research Ethics [61] and therapy was conducted according to the Declaration of Helsinki: Ethical Principles for Medical Research Involving Human Subjects [62]. Participants’ written, informed consent was required to take part in the study. The first consent was limited to three years. After three years, the participants were asked if they wanted to continue taking part in the study, and those who accepted, gave a new, non-time-limited informed and written consent.” Furthermore, under Procedures in the Methods section, the following is specified (with new parts in italic): “Participants were carefully informed, in writing and verbally, about the purpose of the study, about confidentiality, that participation in the study was voluntary, that rejecting was of no consequence for their treatment, and that, if they signed up for the study, they could withdraw at any time. Seventy-two patients were asked to participate in the study, and 54 accepted and gave their written consent (75% inclusion).”*

3. We note that you have indicated that data from this study are available upon request. PLOS only allows data to be available upon request if there are legal or ethical restrictions on sharing data publicly. For information on unacceptable data access restrictions, please see http://journals.plos.org/plosone/s/data-availability#loc-unacceptable-data-access-restrictions.

In your revised cover letter, please address the following prompts:

a) If there are ethical or legal restrictions on sharing a de-identified data set, please explain them in detail (e.g., data contain potentially identifying or sensitive patient information) and who has imposed them (e.g., an ethics committee). Please also provide contact information for a data access committee, ethics committee, or other institutional body to which data requests may be sent.

b) If there are no restrictions, please upload the minimal anonymized data set necessary to replicate your study findings as either Supporting Information files or to a stable, public repository and provide us with the relevant URLs, DOIs, or accession numbers. Please see http://www.bmj.com/content/340/bmj.c181.long for guidelines on how to de-identify and prepare clinical data for publication. For a list of acceptable repositories, please see http://journals.plos.org/plosone/s/data-availability#loc-recommended-repositories.

We will update your Data Availability statement on your behalf to reflect the information you provide.

*We have uploaded the minimal anonymized data set necessary to replicate our study findings as Supporting Information files.*

## **2. Response to reviewer**

*Thank you very much for your time reviewing our manuscript and for helpful comments.*

1. Is the manuscript technically sound, and do the data support the conclusions?

The manuscript must describe a technically sound piece of scientific research with data that supports the conclusions. Experiments must have been conducted rigorously, with appropriate controls, replication, and sample sizes. The conclusions must be drawn appropriately based on the data presented.

Reviewer #1: Partly

*Answers are given below to the specific comments from the reviewer.*

2. Has the statistical analysis been performed appropriately and rigorously?

Reviewer #1: No

*Answers are given below to the specific comments from the reviewer.*

3. Have the authors made all data underlying the findings in their manuscript fully available?

The PLOS Data policy <<http://www.plosone.org/static/policies.action#sharing>> requires authors to make all data underlying the findings described in their manuscript fully available without restriction, with rare exception (please refer to the Data Availability Statement in the manuscript PDF file). The data should be provided as part of the manuscript or its supporting information, or deposited to a public repository. For example, in addition to summary statistics, the data points behind means, medians and variance measures should be available. If there are restrictions on publicly sharing data—e.g. participant privacy or use of data from a third party—those must be specified.

Reviewer #1: No

*We have now uploaded the data underlying our findings* *as Supporting Information files.*

4. Is the manuscript presented in an intelligible fashion and written in standard English?

PLOS ONE does not copyedit accepted manuscripts, so the language in submitted articles must be clear, correct, and unambiguous. Any typographical or grammatical errors should be corrected at revision, so please note any specific errors here.

Reviewer #1: Yes

*Thanks for this positive assessment.*

## **Reviewer’s comments (normal font) and our responses (*in italics*)**

1. Introduction. : I found the introduction, whilst extremely interesting, it was too long and needs to have a clearer path towards the overall aim of this paper. For example, lines 86 to 151 seems to be discussing therapies, but your participants seemed to be provided with a wide variety of treatments from a variety of specialists (e.g. lines 277 to 286). It is unclear that your models measured the nature of the treatment?

*We have tried to shorten and focus the introduction in a better way, as recommended. It is true that our models did not measure the nature of the treatment. We have therefore omitted the discussion of therapy methods in the introduction. We have also omitted the part on whether treatment results in this patient group represents “value for money”, and made some additional adjustments to the introduction, hopefully representing a clearer path towards the aim of the paper. However, we have kept passages that more generally refer to results of therapy for this patient group. We have also kept passages leading up to the importance of longitudinal studies, and passages commenting on how or whether length of treatment is related to treatment outcome. Part of our participants received very long treatments, but they may not have progressed correspondingly.*

2. Sample size: Please perform a power analysis for your mixed methods models (Note: there are a number of R packages that can perform these for you).

*In 2006 we consulted two statisticians about sample size in our study. They said a sample size of 100 would be better, but they gave the opinion that with reasonable effect sizes of expected changes, and repeated measurements, we could still do meaningful analyses with an N of about 50. At that time, we did not have resources to continue including more participants to the study. Aims of the study were multiple, and part of the aims were explorative, since in-depth, longitudinal and multi-method studies of this patient group was largely non-existing at the time – and is still scarce.*

*A formal power analyses was thus not performed at the beginning of our study. A power analysis after data collection is not recommended. We refer to the following from Lydersen (2019): “After the study, it is generally recommended to report an estimate and a 95 % confidence interval for the effect, as well as a p-value. Sometimes, a journal or a reviewer requests a calculation of the ‘observed power’ in addition (*[*2*](https://tidsskriftet.no/en/2019/01/medisin-og-tall/statistical-power-not-after#ref2)*). This means a statistical power calculation based on the observed effect, as if it were a future study, and reporting it as if it gives additional information about the study already performed. This is not only fundamentally flawed, but it gives no information in addition to the reported p-value” (p. 1). Ref.: Stian Lydersen (2019). Statistical power: Before, but not after! Tidsskrift for den norske legeforening (Journal of the Norwegian Medical Association).* (<https://tidsskriftet.no/en/2019/01/medisin-og-tall/statistical-power-not-after>). *This article includes further references with the same conclusion.*

3. Participants: Please report on the proportion from Asia, Europe and Africa as these continents do differ and the reader should understand the mix of your participants.

*The distribution of backgrounds has now been added: “The participants, 35 men and 19 women, came from 15 different countries* *in Asia, Europe, and Africa. Of these, 57.4% (n = 31) came from countries in the Middle East, 16.7% (n = 9) came from other Asian countries, 16.7% (n = 9) came from countries in the Balkans, and 9.3% (n = 5) came from various African countries.*

4. Procedures: It is unclear as to the distribution of timing of termination of therapy as in line 265 you state “…. Termination of therapy, whenever that occurred”.

*In the process of avoiding repetitions in the manuscript, this phrase disappeared. The meaning was, however, “termination of therapy, which occurred after individually varying lengths of therapy”.*

5. Measures: Please provide citations for your clinical cutoff. Also, please ensure you outline to the reader what high scores of your measures mean.

*In the instruments section, references for the clinical cutoff is now provided* *for* *HTQ, part IV and for* *HSCL-25. The meaning of high scores should also now be clear in the description of the instruments.*

Please clarify if these are self-report measures as you reference the DSV-IV but it does not appear to be SCIDs?

*The symptom measures are all based on the instruments used in the study, which are described in the Instruments section. The interviewers (a specialist in clinical psychology, and a specialist in psychiatry/psychoanalyst) generally read the questions/items to the participants, asked follow-up questions if the participant’s answer was unclear, and noted their responses. In the Instruments section, we have added the following: “According to Mollica et al. [58], the HSCL- 25 and the HTQ were not designed as a self-report, but should always be administered to traumatized refugees by health care workers, in an atmosphere of empathy.”*

*In* *the Procedures section of the current manuscript, we have added the words marked in italic:* ***“****As recommended by Mollica et al. [58], the checklists and questionnaires were completed in the presence of the researchers, who offered assistance and explanations when needed, and who listened and responded in an atmosphere of empathy. Most of the participants preferred that the researchers read the questionnaire and checklist items to them and noted their responses.”*

*In the first publication from the study in 2013 [55]), the procedure was outlined in more detail: “All questionnaires were administered personally by the researchers, question by question, and translated, when needed, orally, on the spot. Associations to the questions and exemplifications of the answers were allowed. We asked several follow-up questions before placing a check mark noting the intensity of the problem. These procedures were time consuming, but gave us indications of how the questions were understood and added important information elaborating the fixed choices of answers in the questionnaires. Mollica et al. (2004) recommended that the HSCL-25 and the HTQ always be administered to traumatized refugees by health care workers, in an atmosphere of listening with empathy. They stated that neither instrument was designed as a self-report.”*

Reference to DSM-IV:

*The HTQ instrument is described with reference to DSM-IV in its manual. We have added the full name of the DSM-IV. In our manuscript, under Instruments, we describe the HTQ the following way:* ***“****The Harvard Trauma Questionnaire, Symptoms of PTSD* *(HTQ, Part IV, Questions 1-16) [58], is a symptom checklist consisting of 16 items derived from the criteria for PTSD in the Diagnostic and statistical manual of mental disorders, fourth edition (DSM-IV) [60],…”. So as not to cause any misunderstanding elsewhere in the manuscript, we removed the words* ***“****DSM-IV-based” from other references to the results of the symptom checklists.*

6. Measures: Please provide the relevant research behind your development of the “Daily life function”.

*In the Introduction we have added a passage about the interdependence between mastering the language of the host country, completing education or holding employment and mental health, wellbeing, and of functioning well in exile. Thanks for making us aware of this omission in the introduction. Throughout the text we have changed the expression “daily life function” to “exile life functioning”/” functioning in exile”. We found this to be more precise.*

7. Missing data: Please report on the missing data on both the time invariant and time varying variables.

*Participants’ needs and (lack of) endurance had priority before our needs and plans as researchers. At some follow-ups, participants willingly talked with us on the qualitative parts of the follow-up, but refrained from, or had to leave before responding to one or more of the questionnaires. However, as questionnaires and symptom checklists were conducted in interview form, there were not many missing items within the questionnaires, once they were responded to at all. We conducted around 500 interviews during this longitudinal study, both the number of interviews and their timings varied considerably between participants. It would be too much detail if we were to outline each time we had a follow-up with any of the participants when one or more of the questionnaires were not completed.*

*We have described in general terms in the Procedures section that participants were recruited consecutively over three years, and that they were interviewed at different dates and intervals. In the beginning of the Results section, under Descriptives, we have described how many times each questionnaire was in fact completed, and over what period of time.*

*The way the data was arranged for this analysis, allowed us to see how many times each participant completed each questionnaire, the date for each completion, and across how many years each respondent responded to the questionnaires.*

*The following is now described in the Statistical procedures section,: “To examine changes in mental health symptoms and QOL, including all measures obtained at different times and intervals for the 54 participants over the 10 years of study, we used linear mixed effects (lme). lme is a statistical method suited to analyse repeated measurements in longitudinal studies, even when number of observations and the observation times differ among subjects [67,68]. Time was modelled as continuous from T1. All measures over the entire research period were utilized to estimate the progress. Missing data were left missing. 80 % of the items comprising each variable had to be completed if the variable was to be computed. ”*

8. Table 2: Please include base at each time point and for each measure.

*We do not fully understand this comment. Data was collected at different times and intervals. Please see our comments on point 7.*

Also, your title indicates this is a 10-year study, but Table 2 only reports up to the first 3 years. Please provide the relevant information.

*To avoid confusion of what belongs to the lme, which is the main analysis of the current study, the original Table 2 is removed and merged with Table 1 (Table 1. Demographics, mental health symptoms, quality of life, and indicators of functioning in exile upon entering therapy and the study). It now only shows results from intake – baseline. We have omitted data from T2 and T3, because they are not a result of the lme, but were formerly modelled for an earlier publication, before the data collection was completed. Table 1 is shown in the Methods section. Data from intake/baseline is included in, but not a result of the present study.*

*Furthermore, to ease consistency, we have now changed* *the modelled trajectories in Figures 1 and 2 to show the whole 10-year period, and made sure the results are described and illustrated for 10 years throughout the study.*

*The data for the lme were organized consecutively, including results from each time each questionnaire was completed by each participant, and at what date. In the lme, for each individual, time for each follow-up questionnaire response was calculated from the study intake date to the date of each follow-up.*

9. Diagnostic Evaluation: (Lines 339-350) It is very unclear who coded the ICD-10 diagnoses and at what time period(s) these diagnoses occurred? This is the first time ICD-10 has been mentioned and requires more clarification as these do not appear to be clinical diagnoses so it is unclear as to why they are referred to? ICD-10 is predominantly used for billing purposes and within hospitals for general diagnoses and procedure reporting. Also, please define “ICD-10” to the reader.

*All Norwegian mental health clinics, part of the Norwegian specialized health care system, use ICD-10 (The International Statistical Classification of Diseases and Related Health Problems, 10th Revision, WHO, 1994) as their formal diagnostic system. Maybe reporting of the diagnostic evaluation from the clinics only serves to confuse. To make the manuscript more readily understandable and straight forward, we have omitted information about what diagnoses the participants were given in the treatment unit.*

10. Statistical procedures: You indicate you modelled time “as continuous from T1” (Line 363), but this assumes equal time periods but you have T1 baseline, 1 year, then 3 years, then it is unclear the other time periods.

*In the Results section, under Descriptives, and in the Methods section, the variability in number of follow-ups and variability of intervals between follow-ups among the participants is described. Please also see our response to point 8.*

LME assumes a linear relationship for equal increments and this does not appear to be the case for your data. Did you perform a sensitivity analysis with time as a categorical variable with T1 as base reference? If not, please do so.

*Mixed effects models have the advantage of not assuming equal increments, and the measurement times may vary between participants. The lme modelled linear trajectories of the scores of the symptom clusters and quality of life dimensions. All model assumptions are approximations, but we have now checked non-linearity without finding indications of systematically non-linear trajectories. As illustrations, plots including smooth curves for PTSD, anxiety, depression and the quality of life dimensions are included as Supplemental files. As we have described, and as can be seen on the smooth curves, the results are more uncertain for the last few years, as these included responses from only a few participants, potentially a selected part of the sample.*

11. Statistical procedures: Please include the ICC.

*Our mixed effects models include random variation between persons both for the intercept and the slope, therefore an intraclass correlation is not available based on these models.*

12. Statistical Procedures: In your LME models, did you control for potential time invariant confounders such as continent? What interactions with time did you test?

*There are widely different cultures represented in each continent, so in our view, it will not make sense to control for continents. Moreover, the distribution of participants is not equally dispersed among the continents. This study is based on eligible participants, without regard to national origins, among patients being referred to, and accepted for treatment, as naturally occurring in the Norwegian mental health services that we collaborated with at the time of inclusion (2006-2009). We did not test any possible confounders at this point.*

13. Results: Please provide sample sizes for all your results as it appears much of your results are based on small sample sizes. For example, Changes in Daily Life Functioning have n<10.

*We see that the way this was formerly outlined could be unclear. In Table 1, all N’s are now within the table. Changes in Daily Life Functioning, now termed Exile life functioning, occurred only in 10 or less (n) from intake to the study until our last meeting with each participant, but the investigated N included all of the 54; the ones who changed as well as the rest of the sample who did not change status from intake to our last meeting with them, whether they at intake already held a job and could communicate in Norwegian or not. In the Results section, Changes in Exile life functioning, we have tried to state more clearly the bases for these calculations. Sample sizes throughout the study for the data included in the lme cannot be given for reasons explained under point 7, but the number of completed questionnaires are described under Descriptives in the Results section.*

14. Results: Your graphs extend to 6 years, but your descriptives are up to T3 and the title of your manuscript indicates up to 10 years. Please ensure your time points are consistent for the reader.

*Please see our response to point 8. We hope the manuscript will now appear consistent throughout.*
