# Supplementary material for: The 10-year course of mental health, quality of life, and exile life functioning in traumatized refugees from treatment start
Source: PLoS One. 2020 Dec 31;15(12):e0244730. doi: 10.1371/journal.pone.0244730 (PMC7775068; doi:10.1371/journal.pone.0244730)
Supplement: Attachment — Submitted filename: Response to Reviewers.docx [file pone.0244730.s007.docx]

**Response to Reviewers**

PONE-D-20-12948R1

Note*. Our responses in italics.*

*Thank you again for reviewing our manuscript, our comments to the previous review, and for your new comments.*

**6. Review Comments to the Author**

Reviewer #1: Thank you for answering my review questions, clarifying your research methodology and arguing your stance.

Reviewer #2: A 10-year longitudinal study was conducted to study the changes in symptoms of posttraumatic stress, anxiety and depression in refugee participants receiving psychotherapy. Linear mixed effect models were used to determine changes in quality of life measures. Symptoms, quality of life, and exile life functioning improved over the course of the study.

Minor revisions:

1- There are procedures/macros available outside of the mixed effects models that can be used to calculate intraclass correlation coefficients.

*The question of whether to report an intraclass correlation based on our mixed effects models is not a question of lack of software. All computations involving random effects in our mixed effects models may be done within the estimated models, with no need for external software.*

*As commented in our previous reply, it is not clear what could be the meaning of an intraclass correlation coefficient, in a mixed effects model with between person random variation both in the intercept and the slope. An intraclass correlation is defined in terms of standard deviations for random effects within and between persons, in a mixed effects model with only one random effect within and one random effect between persons. In our models, there is only one random effects within persons, but two random effects between persons.*

2- Indicate the covariance structure used in the linear mixed model and the criteria for selecting it.

*We have included a random effect for within person variation and random effects for variation in intercept and slope between persons. We have not included a further covariance structure for the error terms within persons, and we have not stated that such a structure is part of the model. The reason is that the time points, and the number of time points, varies considerably between subjects. This may be a limitation, but we do not see a possibility for hypothesizing a specific covariance structure for such a case.*

3- Within the manuscript, state and justify the study’s target sample size with a pre-study statistical power calculation for the mixed model.

*As commented in our replies in the previous round, there was not performed a formal sample size calculation when the study was initiated back in 2006. This is a limitation, but there is no way this could be rectified now since post hoc sample size calculations are methodologically flawed, as detailed in references given in our previous reply. We have added the lack of a pre-study power calculation to Limitations, page 28, line 599.*

4- When data is missing, indicate the analyzed sample size.

*Sample sizes and all details of the pretreatment assessment and the assessment of all participants one and three years after inclusion into the study are described in former publications (Opaas and Hartmann, 2013; Opaas and Varvin, 2015; Opaas, Hartman, Wentzel-Larsen, & Varvin, 2016).*

*Except for these three coordinated assessment points, all other assessment points were random, that is, not coordinated according to the time of inclusion into the study for each individual. Therefore, it is not meaningful to speak of missing data and sample size at each point in time. The statistical method chosen is suited for such data, where time between each measurement and the number of assessments varies among the participants.*

*In Procedures, page 13, line number 249 to 252, an addition is made to make the nature of the data clearer.*
